# Association Between Intoxication and Urgent Neurosurgical Procedures in Severe Traumatic Brain Injury: Results From the American College of Surgeons Trauma Quality Improvement Program

**DOI:** 10.1177/08850666211017497

**Published:** 2021-05-20

**Authors:** Bourke W. Tillmann, Avery B. Nathens, Damon C. Scales, Barbara Haas

**Affiliations:** 1 Interdepartmental Division of Critical Care, University of Toronto, Ontario, Canada; 2 Department of Critical Care Medicine, 71545 Sunnybrook Health Sciences Centre, Toronto, Ontario, Canada; 3 Institute of Health Policy, Management, and Evaluation, 7938 University of Toronto, Ontario, Canada; 4 Department of Surgery, 7938 University of Toronto, Ontario, Canada; 5 Sunnybrook Research Institute, 7938 Toronto, Ontario, Canada; 6 Department of Medicine, 7938 University of Toronto, Ontario, Canada; 7 Li Ka Shing Knowledge Institute of St. Michael’s Hospital, Toronto, Ontario, Canada

**Keywords:** craniocerebral trauma, neurosurgical procedures, quality improvement, healthcare disparities, registries

## Abstract

**Background:**

The probability of undergoing surgery after severe traumatic brain injury (TBI) varies significantly across studies and centers. However, causes of this variability are poorly understood. We hypothesized that intoxication may impact the probability of receiving an urgent neurosurgical procedure among patients with severe TBI.

**Methods:**

We performed a retrospective cohort study of adult patients admitted to a Level I or II trauma center in the United States or Canada with an isolated severe TBI (2012–2016). Data were derived from the Trauma Quality Improvement Program dataset. An urgent neurosurgical procedure was defined as a procedure that occurred within 24 hours of admission. Multivariable logistic regression was utilized to examine the independent effect of intoxication on a patient’s likelihood of undergoing an urgent procedure, as well as the timing of the procedure.

**Results:**

Of the 33,646 patients with an isolated severe TBI, 11,313 (33.6%) were intoxicated. An urgent neurosurgical procedure was performed in 8,255 (24.5%) cases. Overall, there was no difference in the probability of undergoing an urgent procedure between patients who were and were not intoxicated (OR 0.99; 95% CI 0.94–1.06). While intoxication status had no impact on the probability of surgery among patients with the most severe TBI (head AIS 5: OR 1.06 [95% CI 0.98–1.15]), intoxicated patients on the lower spectrum of injury had lower odds of undergoing an urgent procedure (AIS 3: OR 0.80 [95% CI 0.66–0.97]). Among patients who underwent an urgent procedure, intoxication had no impact on timing.

**Conclusion:**

Intoxication status was not associated with differences in the probability of undergoing an urgent neurosurgical procedure among all patients with a severe TBI. However, in patients with less severe TBI, intoxication status was associated with decreased likelihood of receiving an urgent intervention. This finding underscores the challenge in the management of intoxicated patients with TBI.

## Background

Traumatic brain injury (TBI) is the direct or underlying cause of death in one-third of all injury-related fatalities.^1,2^ Although treatment options for severe TBI are limited, in select patients surgical intervention confers a mortality benefit.^3–10^ Consequently, the Brain Trauma Foundation (BTF) has published guidelines regarding indications for surgical intervention in severe TBI.^3,8,11–14^ Nevertheless, even after adjusting for differences in injury type and severity, the frequency of neurosurgical intervention following TBI varies significantly across patient groups and hospitals.^4–6^

Variation in rates of neurosurgical intervention across centers has led to concerns regarding variation in quality of care across hospitals.^4,5,15^ The causes of the observed variation in practice are incompletely understood. Several BTF recommendations are supported by low grade evidence, which likely contributes to variable adherence to these guidelines.^4,5^ In addition, prior studies focused on variation in neurosurgical interventions across centers may have incompletely evaluated differences in patient-level factors. Failure to account for key patient characteristics may have led to the attribution of variations in neurosurgical interventions to differences in hospital practices, when in fact the variation is related to differences in the patient population. Specifically, we hypothesized that variations in the rate of alcohol intoxication among patients with severe TBI contribute to the observed variations in the receipt of neurosurgical procedures.

Up to half of all patients hospitalized with a traumatic injury are intoxicated at the time of the event.^16,17^ Consequently, in many cases of TBI, the initial examination is confounded by the presence of alcohol.^17–19^ The combination of alcohol use disorder and traumatic injury may also be a surrogate marker for marginalization, which itself is a risk factor for decreased access to care.^20–24^ As a result of both a confounded clinical exam and potential disparities in care, patients who present with intoxication and severe TBI may be less likely to undergo urgent neurosurgical intervention. The objective of this study was to explore the association between intoxication status and receipt of neurosurgical intervention in adult patients who sustained a severe TBI.

## Methods

### Study Design and Setting

We conducted an observational cohort study of adult patients admitted to an American College of Surgeons (ACS) Trauma Quality Improvement Program (TQIP) hospital who sustained an isolated, severe TBI secondary to a blunt mechanism between January 1, 2012, and December 31, 2016. The objective of this study was to determine whether a patient’s intoxication status was independently associated with a difference in the probability of undergoing an urgent neurosurgical intervention following severe TBI. This study was approved by the research ethics board of the Sunnybrook Research Institute.

### Data Source

Patient and institution-level data were extracted from the TQIP database. As of 2016, 463 ACS or state designated level I and II trauma centers across the United States and Canada submitted data to TQIP.^25–27^ All patients admitted to a TQIP participating hospital who sustain a severe injury (defined as an injury with a score on the Abbreviated Injury Scale [AIS] ≥ 3) are included in the database.^25^ TQIP contains detailed patient and hospital variables including demographics, comorbid illnesses, injury mechanism and severity, pre-hospital and emergency department (ED) vital signs, in-hospital procedures, discharge disposition, hospital size and type, trauma center status, and surgical support.^27^ Trained data abstractors are employed at each contributing center and data reliability is ensured through standardized education and stringent approaches to data validation.^25,28^

### Study Population

Patients ≥ 16 years of age admitted with an isolated, severe TBI secondary to a blunt mechanism were included. A severe TBI was defined as an AIS score > 3 in the head region, with a presenting score on the motor component of Glasgow Coma Scale (GCS) ≤ 5 and a total GCS ≤ 8. These criteria were specified in order to identify patients who met the BTF criteria for intracranial pressure (ICP) monitoring and were therefore candidates for at least one neurosurgical intervention.^3,4^ Patients with severe multisystem injuries (AIS ≥ 3 in any other body region) were excluded, as management of other injuries might modify the timing and utilization of neurosurgical interventions.^29^ Patients with non-survivable TBI (head AIS = 6) were also excluded. We further excluded patients coded as dead on arrival, those who died in the ED, and those missing data for age, sex, GCS, in-hospital procedures, or intoxication status. Patients > 89 years of age were assigned an age of “-99” in the database and consequently were excluded from the study.

### Identification of Intoxication Status

In TQIP, intoxication status is coded as 1 of 5 mutually exclusive categories; positive above the legal limit (based on the legal limit for blood alcohol concentration in the region where treatment occurred), positive below the legal limit, not tested but assumed to be intoxicated, not tested and assumed to not be intoxicated, or tested negative. To better reflect clinical decision-making, intoxication status was dichotomized as either intoxicated or not intoxicated. Patients coded as “positive above the legal limit”, “positive below the legal limit”, and “not tested but assumed to be intoxicated” were classified as intoxicated, while patients coded as “not tested and assumed to not be intoxicated” and “tested negative” were classified as not intoxicated. As there are significant challenges in the clinical determination of intoxication status, we also performed a sensitivity analysis comparing only patients classified as “positive above the legal limit” to those classified as “tested negative”.^18^

### Outcome Measures

The primary outcome of interest was the receipt of an urgent neurosurgical intervention, defined as a neurosurgical procedure within 24-hours of admission. A neurosurgical procedure was identified by the presence of an International Statistical Classification of Diseases Procedure Coding System (ICD-PCS) code for any of the following procedures: placement of an ICP monitor, craniotomy, or craniectomy (Supplemental Digital Content, Table 1). ICD-9-PCS codes were used to identify interventions for the years 2012 to 2015 and ICD-10-PCS codes were used for the years 2015 to 2016.^4,30^

**Table 1.** Patient and Hospital Characteristics.

	Overall (n = 33,646)	Intoxicated (n = 11,313)	Not intoxicated (n = 22,333)	Standardized difference
Patient characteristics				
Age, median (IQR)	51 (31 – 68)	42 (28 – 55)	53 (33 – 73)	0.56
Age group, n (%)				
16-40	12,478 (37.1)	5,412 (47.8)	7,066 (31.6)	0.66
41-64	11,326 (33.7)	4,657 (41.2)	6,669 (29.9)	
65-89	9,824 (29.3)	1,244 (11.0)	8,598 (38.5)	
Female, n (%)	9,438 (28.1)	2,068 (18.3)	7,370 (33.0)	0.34
Race, n (%)				
Non-Hispanic white	22,683 (67.4)	7,153 (63.2)	15,530 (69.5)	0.15
Hispanic or Latino	3,806 (11.3)	1,539 (13.6)	2,267 (10.2)	
Black	3,524 (10.5)	1,384 (12.2)	2,140 (9.6)	
Other minority group	2,417 (7.2)	852 (7.5)	1,565 (7.0)	
No. Comorbid illnesses, n (%)				
0	11,440 (34.0)	3,848 (34.0)	7,592 (34.0)	0.06
1	11,251 (33.4)	3,828 (33.8)	7,423 (33.2)	
2	6,177 (18.4)	2,089 (18.5)	4,088 (18.3)	
≥ 3	4,778 (14.2)	1,548 (13.7)	3,230 (14.5)	
Type of insurance, n (%)				
Commercial	10,642 (31.6)	3,938 (34.8)	6,704 (30.0)	0.35
Non-commercial	15,580 (46.3)	4,115 (36.4)	11,465 (51.3)	
Self-pay	4,569 (13.6)	2,167 (19.2)	2,402 (10.8)	
Other	1,233 (3.7)	495 (4.4)	738 (3.3)	
Intoxication category, n (%)				
Confirmed not intoxicated	14,005 (41.6)	0 (0)	14,005 (62.7)	1.00
Not tested assumed not intoxicated	8,328 (24.8)	0 (0)	8,328 (37.3)	
Not tested assumed intoxicated	843 (2.5)	843 (7.5)	0 (0)	
Intoxicated below the legal limit	2,083 (6.2)	2,083 (18.4)	0 (0)	
Intoxicated above the legal limit	8,387 (24.9)	8,387 (74.1)	0 (0)	
Injury characteristics				
Mechanism of injury, n (%)				
Fall	17,441 (51.8)	4,815 (42.6)	12,626 (56.5)	0.32
MVC	5,614 (16.7)	2,022 (17.9)	3,592 (16.1)	
Motorcycle	2,037 (6.1)	872 (7.7)	1,165 (5.2)	
Pedestrian	2,004 (6.0)	764 (6.8)	1,240 (5.6)	
Cyclist	997 (3.0)	308 (2.7)	689 (3.1)	
Other	5,372 (16.0)	2,484 (22.0)	2,888 (12.9)	
AIS head, n (%)				
3	6,613 (19.7)	2,710 (24.0)	3,903 (17.5)	0.24
4	11,491 (34.2)	4,179 (36.9)	7,312 (32.7)	
5	15,542 (46.2)	4,424 (39.1)	11,118 (49.8)	
Type of head injury, n (%)				
EDH	3,012 (9.0)	1,116 (9.9)	1,896 (8.5)	0.05
SDH	20,290 (60.3)	6,401 (56.6)	13,889 (62.2)	0.11
Traumatic SAH	16,184 (48.1)	5,805 (51.3)	10,379 (46.5)	0.10
Intracerebral mass lesion	10,838 (32.2)	3,628 (32.1)	7,210 (32.3)	<0.01
Compressed basal cisterns	2,026 (6.0)	607 (5.4)	1,419 (6.4)	0.04
Brainstem/cerebellar lesion	3,402 (10.1)	1,122 (9.9)	2,280 (10.2)	<0.01
Other brain injury without any of the above	3,486 (10.4)	1,457 (12.9)	2,029 (9.1)	0.12
ISS, median (IQR)	24 (16 – 26)	20 (16 – 26)	24 (16 – 26)	0.14
ISS, n (%)				
9-15	5,420 (16.1)	2,195 (19.4)	3,225 (14.4)	0.21
16-24	12,680 (37.7)	4,693 (41.5)	7,987 (35.8)	
25-75	15,546 (46.2)	4,425 (39.1)	11,121 (49.8)	
Minor injury in another body region, n (%)	19,062 (56.7)	7,236 (64.0)	11,826 (53.0)	0.22
Hypotension (sbp <90), n (%)	1,302 (3.9)	445 (3.9)	857 (3.8)	<0.01
Motor gcs, n (%)				
1	22,090 (65.7)	7,491 (66.2)	14,599 (65.4)	0.07
2-3	3,228 (9.6)	909 (8.0)	2,319 (10.4)	
4-5	8,053 (23.9)	2,815 (24.9)	5,238 (23.5)	
Hospital characteristics				
Teaching status, n (%)				
University	19,124 (56.8)	6,229 (55.1)	12,895 (57.7)	0.04
Community	11,590 (34.5)	4,102 (36.3)	7,488 (33.5)	
Non-teaching	2,932 (8.7)	982 (8.7)	1,950 (8.7)	
Bed size, n (%)				
≤ 200	1,004 (3.0)	492 (4.4)	512 (2.3)	0.16
201-400	7,158 (21.3)	2,749 (24.3)	4,409 (19.7)	
401-600	10,088 (30.0)	3,303 (29.2)	6,785 (30.4)	
> 600	15,396 (45.8)	4,769 (42.2)	10,627 (47.6)	
Trauma center level, n (%)				
I	23,244 (69.1)	7,604 (67.2)	15,640 (70.0)	0.07
II	9,811 (29.2)	3,466 (30.6)	6,345 (28.4)	
Other	591 (1.8)	243 (2.2)	348 (1.6)	
No. Neurosurgeons, n (%)				
≤ 2	2,433 (7.2)	964 (8.5)	1,469 (6.6)	0.13
3-5	15,159 (45.1)	5,442 (48.1)	9,717 (43.5)	
≥ 6	16,054 (47.7)	4,907 (43.4)	11,147 (49.9)	

Abbreviations: IQR, interquartile range; AIS, Abbreviated Injury Scale; EDH, epidural hematoma; SDH, subdural hematoma; SAH, subarachnoid hemorrhage; ISS, Injury Severity Score; SBP, systolic blood pressure; GCS, Glasgow Coma Scale; MVC, motor vehicle collision.

The secondary outcome of interest was ‘timeliness’ of the urgent neurosurgical procedure. To define timeliness, we identified the 75th time percentile for receipt of an urgent neurosurgical procedure among the cohort of patients who received a neurosurgical procedure within 24 hours of hospital admission. Any patient whose procedure occurred at, or prior to, the 75th percentile was considered to have received a timely procedure. If a patient received more than 1 neurosurgical procedure, only the first neurosurgical procedure was used to calculate time to receipt of a procedure.

### Relationship Between Intoxication and Urgent Neurosurgical Procedures

We developed a multivariable logistic regression model to evaluate the independent association between intoxication status and the receipt of an urgent neurosurgical procedure, as well as separate multivariable logistic regression model for each specific procedure (placement of an ICP monitor, craniotomy, and craniectomy). Variables assessed for inclusion in the model encompassed baseline characteristics that would be available to clinicians deciding whether to proceed with a neurosurgical intervention. These variables included patient level factors (age, sex, race, intoxication status, insurance status, and comorbid illnesses) and injury characteristics (year, mechanism, Injury Severity Score [ISS], AIS for the head region, presence of a minor injury [AIS ≤ 2] in another body region, initial systolic blood pressure, initial total GCS, initial motor GCS, and type of intracranial lesion). Intracranial lesions were identified using AIS predot codes (1998 version) that reflected injuries to intracranial structures (Supplemental Digital Content, Table 2). Given the relationship between hypotension and poor outcomes among patients with TBI, the initial systolic blood pressure was dichotomized as either ≥ 90 mmHg or below 90 mmHg.^31^ To appropriately adjust for the relationship between age and receipt of an urgent intervention, the model included an interaction term between age and whether a patient was above or below age 65. That is to say, an increase in 1 year of age had a different impact on the likelihood that an older adult would undergo an urgent intervention as compared to a younger adult. Additionally, hospital characteristics (size, trauma center level, and number of neurosurgeons) were also assessed for inclusion in the model.

**Table 2.** Unadjusted Outcomes, Overall and by Intoxication Status.

	Overall (n = 33,646)	Intoxicated (n = 11,313)	Not Intoxicated (n = 22,333)	*P*-Value
Receipt of a neurosurgical procedure, n (%)				
Any point during hospitalization	9,810 (29.2)	3,395 (30.0)	6,415 (28.7)	0.01
Urgent procedure	8,255 (24.5)	2,878 (25.4)	5,377 (24.1)	0.006
Type of urgent procedure, n (%)^a^				
ICP monitor	4,644 (56.3)	1,717 (59.7)	2,927 (54.4)	<0.001
Craniotomy	3,798 (46.0)	1,219 (42.4)	2,579 (48.0)	<0.001
Craniectomy	1,887 (22.9)	703 (24.4)	1,184 (22.0)	0.01
Timing of urgent procedure				
Time to first urgent procedure (hours), median (IQR)	2.42 (1.70 – 4.00)	2.53 (1.80 – 4.13)	2.35 (1.65 – 4.00)	<0.001
Receipt of an urgent procedure within 4 hours of admission, n (%)	6,223 (75.4)	2,135 (74.2)	4,088 (76.0)	0.06
Hospital outcomes				
Died in hospital, n (%)	10,864 (32.3)	2,638 (23.3)	8,226 (36.8)	<0.001
LOS (days), median (IQR)	7 (3 – 16)	8 (3 – 18)	7 (3 – 16)	<0.001
LOS (days) among survivors, median (IQR)	11 (5 – 21)	11 (5 – 21)	11 (6 – 21)	<0.001

Abbreviations: Urgent procedure, within 24 hours of admission; IQR, interquartile range; ICP, intracranial pressure; LOS, length of stay.

^a^ As patients could receive more than 1 urgent neurosurgical procedure total number greater than number of patients who received an urgent neurosurgical procedure.

All variables were assessed for multicollinearity. Any variable found to have a variance inflation factor (VIF) ≥5.0 was considered collinear. Variables considered to be of lesser clinical significance were removed sequentially until no variables with a VIF ≥5 remained. The remaining variables were assessed for inclusion in the model based on their association with a neurosurgical intervention and their role in the causal pathway.^32–34^

A priori we hypothesized that motor GCS and the severity of the head injury may be important effect modifiers in the relationship between intoxication status and receipt of an urgent neurosurgical intervention.^19^ To test this hypothesis, 2 pre-specified subgroup analyses for motor GCS and AIS head were performed. If the interaction term between intoxication and the subgroup was found to be statistically significant the term was kept in the model.

To evaluate the independent relationship between intoxication status and the odds of receiving an urgent neurosurgical procedure in a timely manner, we created an additional multivariable logistic regression model. The model was developed using the same steps as above. In this model, there was no interaction between change in age and age group. Consequently, the interaction term was removed from the model and age was treated as a continuous variable. The analysis of the secondary outcome was limited to those patients who received an urgent neurosurgical procedure. If a patient received more than 1 neurosurgical procedure, only the first neurosurgical procedure was included in the analysis.

### Statistical Analysis

Descriptive statistics were used to display baseline characteristics. Continuous variables were presented as medians and interquartile ranges (IQR) and categorical variables were presented as counts and percentages. Standardized differences were calculated to compare baseline characteristics between patients who were and were not intoxicated.

Neurosurgical procedures and hospital outcomes were then compared between patient groups. Chi-square tests were used to compare categorical variables and the Kruskal-Wallis Test were used to compare continuous variables. Multivariable logistic regression was used to determine the independent association (odds ratio [OR]; 95% confidence intervals [CI]) of intoxication on the primary and secondary outcomes.

All statistical analyses were done using SAS version 9.4 (SAS Institute Inc., Cary, NC) with the type I error probability set to 0.05 and standardized differences of > 0.10 considered statistically significant.^35^

## Results

**Figure 1.**
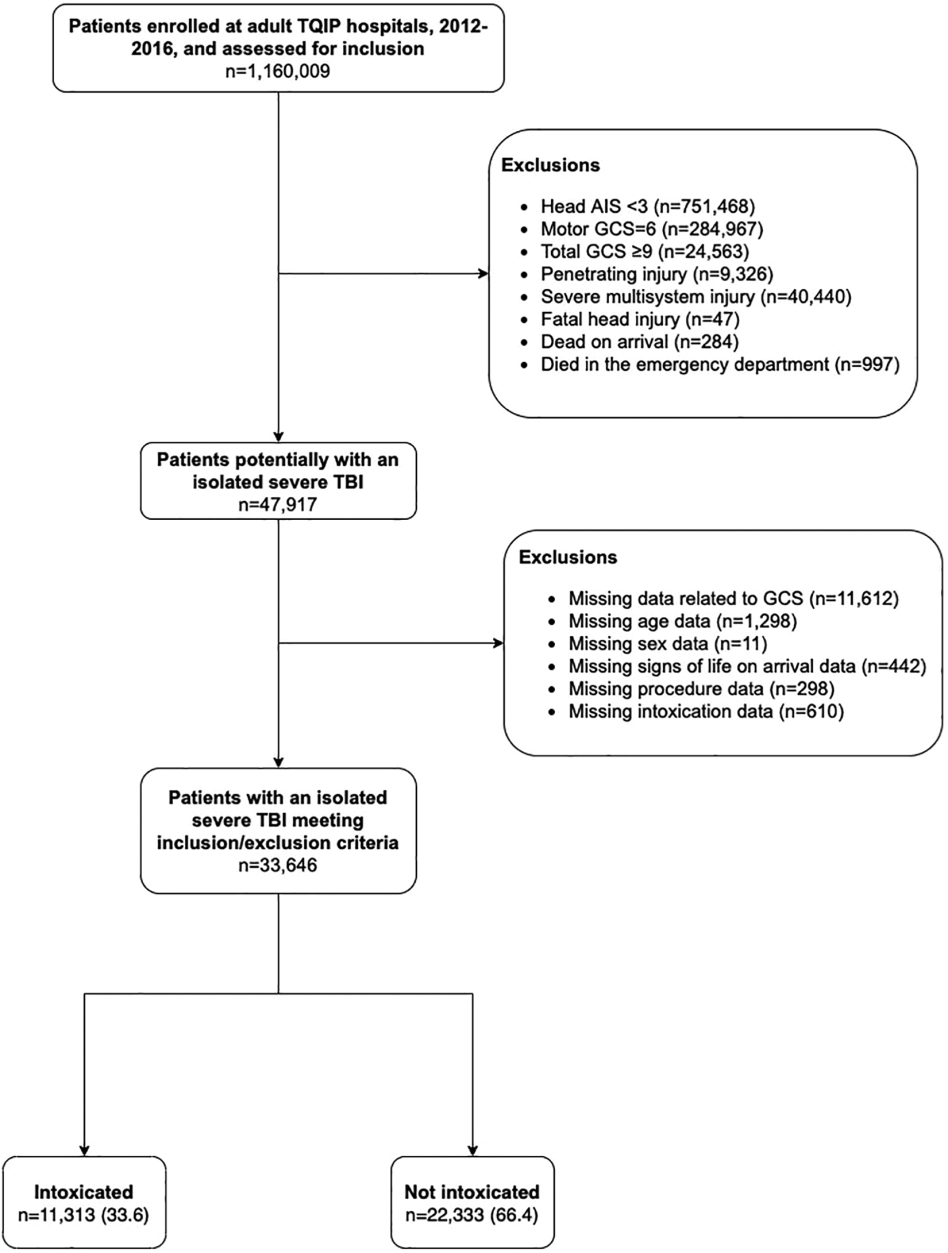
Cohort creation.

During the 5-year study period a total of 33,646 patients met inclusion criteria (Figure 1). Overall, 11,313 (33.6%) were intoxicated, with the majority of these patients (74.1%) having a blood alcohol concentration above the legal limit. Patients who were intoxicated were younger, more often male, more often from a minority group, and more likely to be uninsured (Table 1). Intoxicated patients tended to have less severe head injuries and a greater proportion had an associated minor injury in another body region. Additionally, a greater proportion of intoxicated patients were treated at smaller hospitals with access to fewer neurosurgeons.

The majority of patients (70.8%) did not receive a neurosurgical procedure during their hospital stay (Table 2). Among those who did, 84.1% of the neurosurgical procedures occurred within the first 24 hours, and 75% of these urgent procedures occurred within 4 hours of admission. Patients who received an urgent neurosurgical procedure were younger, more likely to have commercial insurance, less likely to have been injured in a fall, had head injuries of greater severity, and a greater proportion had an epidural or subdural hematoma (Supplemental Digital Content, Table 3).

In unadjusted analyses, a greater proportion of intoxicated patients underwent an urgent neurosurgical procedure as compared to patients who were not intoxicated (Table 2). There was also a significant difference in the type of urgent procedure performed among patients who were or were not intoxicated; patients identified as intoxicated were more likely to have an ICP monitor placed or undergo a craniectomy whereas patients who were not intoxicated were more likely to undergo a craniotomy. Although there was a small increase in the time to first urgent procedure between patients who were and were not intoxicated, there was no difference in the proportion of patients within each group who underwent an urgent procedure within 4 hours of hospital admission.

### Risk-Adjusted Relationship Between Intoxication and Receipt of a Neurosurgical Procedure

ISS and total GCS were not included in the final multivariable logistic regression model to measure the relationship between intoxication status and receipt of an urgent neurosurgical procedure, as they were found to be collinear with AIS and motor GCS. The remainder of the clinically relevant variables were retained in all models with sufficient degrees of freedom (Supplementary Digital Content, Tables 4–7).

**Figure 2.**
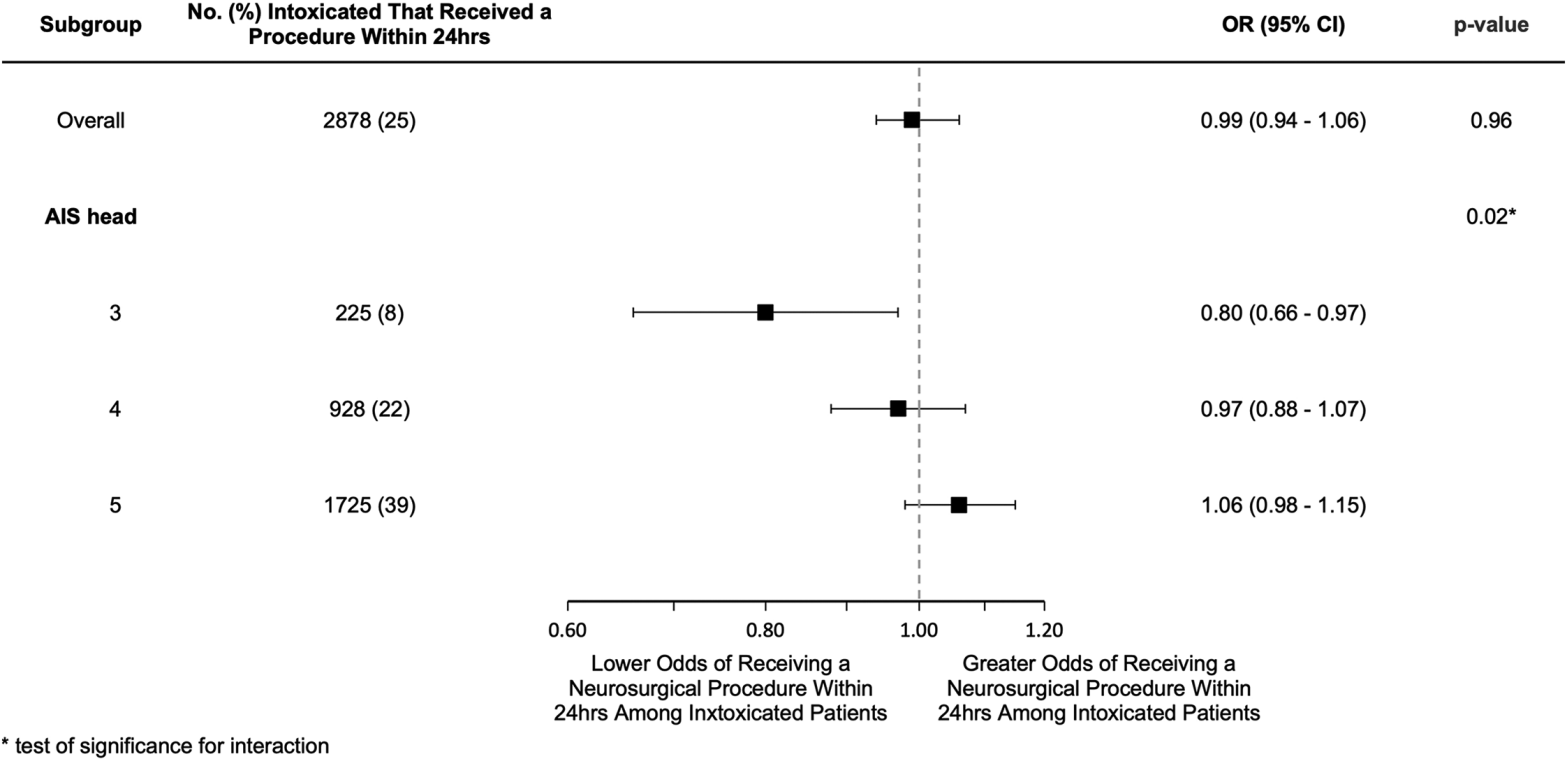
Adjusted odds ratios for receipt of an urgent neurosurgical procedure.

After adjustment for clinically relevant confounders, there was no independent relationship between intoxication status and receipt of an urgent neurosurgical procedure (OR 0.99; 95% CI 0.94-1.06). In the pre-specified subgroup analysis, the relationship between intoxication status and receipt of an urgent neurosurgical procedure was modified by the severity of the head injury (test for interaction, *P* = 0.02), (Figure 2). Specifically, among patients with an AIS of 3, intoxicated patients had lower odds of receiving an urgent neurosurgical procedure compared to patients who were not intoxicated (OR 0.80; 95% CI 0.66-0.97). As the severity of the head injury increased, the impact of intoxication status on receipt of an urgent neurosurgical intervention was attenuated. There was no interaction between intoxication and the patient’s motor GCS score.

The trends in the relationship between intoxication status and receipt of an urgent neurosurgical procedure were similar in the analyses of each specific neurosurgical procedure (Supplementary Digital Content, Table 5).

**Figure 3.**
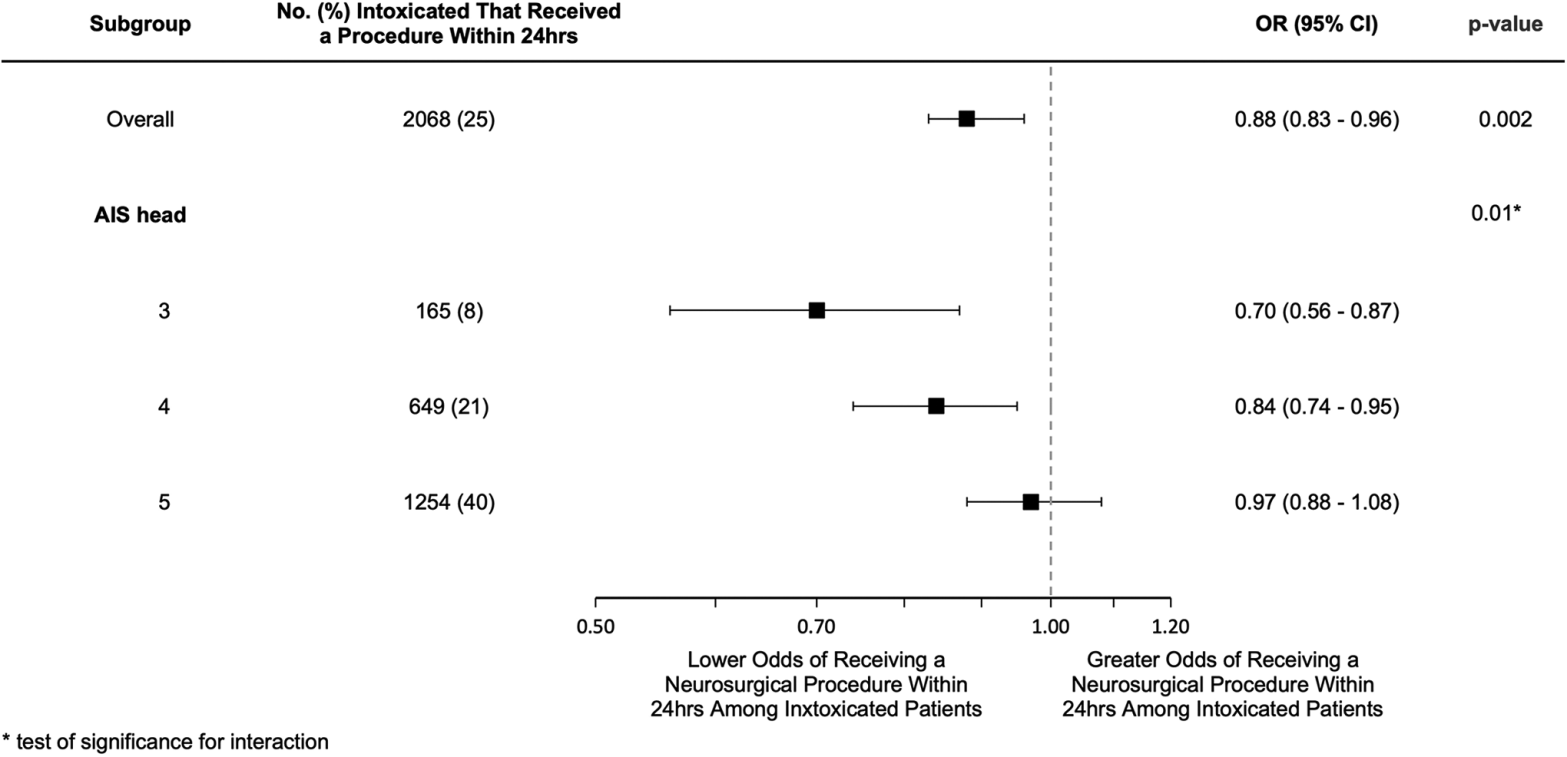
Sensitivity analysis for impact of intoxication on the receipt of an urgent neurosurgical procedure limited to patients confirmed to be intoxicated above the legal limit compared to those who tested negative.

A total of 22,392 patients were included in the sensitivity analysis comparing patients with a blood alcohol concentration above the legal limit to those who had a negative alcohol screen. Of these patients, 25.2% underwent an urgent neurosurgical intervention. Multivariable logistic regression demonstrated that intoxication above the legal limit was associated with lower odds of receiving an urgent neurosurgical procedure (OR 0.89; 95% CI 0.83 – 0.96), (Figure 3, Supplementary Digital Content, Table 6). The severity of the head injury remained an important modifier in the relationship between intoxication status and the receipt of an urgent neurosurgical procedure. In patients with an AIS of 3 or 4, intoxication above the legal limit was associated with lower odds of receiving an urgent neurosurgical procedure (ORs 0.70; 95% CI 0.56-0.87 and 0.84; 95% CI 0.74-0.95 respectively), whereas in patients with an AIS of 5 there was no association between intoxication above the legal limit and the receipt of an urgent neurosurgical procedure (OR 0.97; 95% CI 0.88-1.08).

### Relationship Between Intoxication Status and Timely Receipt of an Urgent Neurosurgical Procedure

After adjustment for clinically relevant confounders, intoxication status was not associated with the timing of an urgent neurosurgical procedures (OR 0.98; 95% CI 0.87-1.10), (Supplementary Digital Content, Table 7). Neither motor GCS nor severity of head injury were identified as important effect modifiers. Examination of each type of procedure separately demonstrated no major differences in the timely receipt of a specific urgent neurosurgical procedure (Supplementary Digital Content, Table 5).The sensitivity analysis restricted to patients with a blood alcohol concentration above the legal limit and those who had a negative alcohol screen also demonstrated no association between intoxication status and the timing of an urgent neurosurgical procedures (OR 0.93; 95% CI 0.81-1.07), (Supplementary Digital Content, Table 6).

## Discussion

Our data suggest that, overall, intoxication is not associated with variation in the probability of undergoing neurosurgical interventions among patients with severe TBI. However, among patients whose anatomical injuries fall on the lower end of the spectrum of “severe injuries”, intoxication appears to be associated with decreased odds of undergoing an urgent neurosurgical intervention. Moreover, significantly elevated blood alcohol concentration is associated with a reduced probability of undergoing surgery among all patients with severe TBI, even after adjusting for other patient and injury characteristics.

Previous studies examining sources of guideline non-compliance regarding the management of traumatically injured patients have identified that treating physicians rely heavily on heuristic cognitive processes.^36,37^ Physicians use pattern recognition to aid in decision making when treating complex patients. It has been postulated that the stigma associated with intoxication may lead to unconscious bias and result in physicians concluding that the presence of intoxication fully explains the patient’s clinical status.^38,39^ Consequently, intoxication may increase a physician’s decisional threshold for obtaining additional investigations and/or proceeding with surgical intervention. The hypothesis that intoxication modifies a clinician’s threshold to intervene, is supported by the variation in the effect of intoxication across strata of head injury severity observed in this study. While intoxicated patients with “less severe” severe TBIs had lower odds of receiving an urgent neurosurgical procedure, intoxication had no impact on the likelihood of undergoing an urgent procedure among patients with the most severe TBIs. This finding suggests that intoxication status plays a greater role in marginal cases, in which current guidelines may be less prescriptive in management decisions.

Although intoxication plays a major role in the occurrence of trauma, its impact on patient outcomes in TBI is controversial.^16^ Low to moderate blood alcohol concentrations have been shown to be associated with improved outcomes in severe TBI, while high blood alcohol concentrations are associated with increased mortality.^17^ One possible explanation for these conflicting findings is a dose dependent neuroprotective effect attributed to alcohol.^40^ Our findings, that the association between intoxication and a decreased likelihood of undergoing an urgent neurosurgical intervention was most evident among those with a blood alcohol concentration above the legal limit provides an additional explanation for the relationship between degree of intoxication and mortality. Simply put, given that there appears to be a dose dependent relationship between intoxication and the likelihood of surgical intervention, any harm from lack of intervention would be concentrated among those least likely to receive the intervention, i.e. those with the highest degree of intoxication.

Due to a lack of access to hospital identification data we were unable to examine variation in rates of urgent neurosurgical interventions across hospitals, or the impact that intoxication status has on this variability. Nonetheless, our findings, that the impact of intoxication is limited either to patients with “less severe” severe TBI, or to those with the highest blood alcohol concentrations, suggests that intoxication itself is unlikely to play a significant role in the variation in inter-hospital rates of neurosurgical intervention. Instead, previous studies suggest that disparities in surgical intervention are related to socioeconomic factors, such as a patient’s race or ethnicity, financial barriers, and systematic policies resulting in lower quality of care.^41–43^ However, given the significant impact that socioeconomic factors have on a patient’s likelihood of undergoing a surgical procedure it is plausible that intoxication may still have an impact on a patient’s likelihood of undergoing an urgent neurosurgical procedure. Instead of confounding the clinical exam, it may be that intoxication represents another surrogate marker for marginalization.^20,21^ Consequently, hospitals which treat a large proportion of intoxicated patients may face the same challenges as hospitals that treat a significantly volume of disenfranchised patients.^44^

Our study has several important limitations. First, only 25% of the cohort underwent an urgent neurosurgical procedure and a majority of the cohort had a motor GCS score of one suggesting that patients may have received sedating medication. However, the inclusion criteria were applied equally to both intoxicated and non-intoxicated patients and therefore the potential inclusion of patients who were not at risk for undergoing a neurosurgical procedure represents non-differential bias and should not alter the results. It is also possible that these low rates of intervention relate to the validity of the codes used to identify surgical procedures. Although these codes have been used in previous studies of TBI and stroke, not all of the codes have been formally validated.^4,30^ However, previous validation of ICD based data to identify in-hospital procedures has demonstrated that procedure codes consistently have high reliability .^45,46^ Likewise, the TQIP utilizes standardized education and stringent approaches to optimize data validation.^25,28^ Furthermore, the rate of neurosurgical intervention identified in this study is similar to previous database studies as well as surveys of clinical practice.^4,6,47^ A second limitation of this study was the inability to account for additional, time varying clinical characteristics, such as changes in hemodynamic parameters or brainstem reflexes including pupillary response. These factors are not captured in TQIP data and therefore our analysis is unable to account for their impact on the decision to proceed with neurosurgical intervention. It is unlikely that there would be a relationship between intoxication status and brainstem reflexes, suggesting that lack of inclusion of this data does not alter the direction of the relationship identified in our study. Another limitation is the possibility that patients who were intoxicated may have systematically been more likely to be identified as having a severe TBI as intoxication would bias them to have a lower GCS score. Therefore, intoxicated patients in this study may have less severe anatomic injuries for a given GCS. We attempted to control for the impact of systematically different GCS scores through the inclusion of the motor GCS score in the multivariable model. Furthermore, the findings of our sensitivity analysis suggest that the impact of intoxication status on the receipt of an urgent neurosurgical intervention extends to patients with more severe TBIs; injuries in which it is likely both intoxicated and non-intoxicated patients would have a score on the motor component of GCS of less than 5. This analysis suggests that our findings are not the result of selection bias. Instead, when examining a more homogenous cohort, the impact of intoxication on the receipt of an urgent procedure is exaggerated and extends to head injuries with an AIS score of 4. A fourth limitation is our lack of access to hospital identification data. Accordingly, we were unable to account for the impact of clustering within hospitals on a patient’s odds of receiving an urgent neurosurgical procedure. Previous studies suggest that hospital factors may play a role in the disparities in surgical outcomes.^35,43,48^ We therefore included general hospital characteristics in our analyses in an attempt to limit the variation in the receipt of a neurosurgical procedure associated with differences across hospitals. Likewise, we included trauma centers from both the United States and Canada. It is possible that differences in the healthcare systems may also impact the receipt of an urgent neurosurgical procedure. Most notably, in Canada all medically necessary in-hospital services are publicly funded with standardized reimbursement rates.^49^ Consequently, the impact of payer status and/or financial incentives may have been attenuated at Canadian trauma centers. To control for the impact of payer status, a patient’s health insurance type was included in the model. Furthermore, trauma systems in Canada and the United States developed in a similar fashion with significant overlaps in organization between the 2 counties such that it is unlikely that substantive differences in injury care exist between the 2 countries.^50,51^ An additional limitation of this study relates to the composite nature of the primary outcome. An urgent neurosurgical procedure was identified as either placement of an ICP monitor, craniotomy, or craniectomy. Whereas craniotomies and craniectomies are considered lifesaving interventions, insertion of an ICP monitor may be viewed as a diagnostic tool with unclear impact on patient outcomes. However, although limited by sample size, subgroup analyses demonstrated consistent trends in the relationship between intoxication and receipt of an urgent procedure across all interventions. Our study was also limited by missing data. Nearly a quarter of patients who potentially had an isolated severe TBI were missing GCS data. Due to the utilization of GCS data in the identification of the patient population, statistical techniques to estimate GCS were not employed. A final limitation of this study was the inability to examine the impact of intoxication or delayed neurosurgical intervention on hospital outcomes. Unmeasured confounders play a significant role in patient outcomes after major trauma and the analyses utilized in this manuscript were not designed to account for such confounding.^4,52^ However, previous studies of severe TBI have demonstrated that high levels of intoxication are associated with worse outcomes, while higher rates of ICP monitor utilization, and management of patients with severe TBI in accordance with evidence-based guidelines are associated with improved outcomes.^4,7,17,53,54^

## Conclusions

In this study we demonstrated that intoxication status was not associated with differences in the probability of undergoing an urgent neurosurgical procedure among all patients who sustain a severe TBI. However, in those patients with less severe TBI or a high blood alcohol concentration, intoxication status was associated with decreased odds of receiving an urgent neurosurgical intervention. These findings may represent a potential mechanism for the differences in outcomes observed across patients with high blood alcohol concentrations. They also underscore the challenge in the management of intoxication patients who have sustained a TBI.

## Supplemental Material

Supplemental Material, sj-pdf-1-jic-10.1177_08850666211017497 - Association Between Intoxication and Urgent Neurosurgical Procedures in Severe Traumatic Brain Injury: Results From the American College of Surgeons Trauma Quality Improvement Program Click here for additional data file. Supplemental Material, sj-pdf-1-jic-10.1177_08850666211017497 for Association Between Intoxication and Urgent Neurosurgical Procedures in Severe Traumatic Brain Injury: Results From the American College of Surgeons Trauma Quality Improvement Program by Bourke W. Tillmann, Avery B. Nathens, Damon C. Scales and Barbara Haas in Journal of Intensive Care Medicine

## Abbreviations

ACS: American College of Surgeons
AIS: Abbreviated Injury Scale
BTF: Brain trauma foundation
CI: Confidence interval
ED: Emergency department
EDH: Epidural hematoma
GCS: Glasgow Coma Scale
ICD-PCS: International Statistical Classification of Diseases Procedure Coding System
ICP: Intracranial pressure
IQR: Interquartile range
ISS: Injury Severity Score
LOS: Length of stay
MVC: Motor vehicle collision
OR: Odds ratio
SAH: Subarachnoid hemorrhage
SBP: Systolic blood pressure
SDH: Subdual hematoma
TBI: Traumatic brain injury
TQIP: Trauma Quality Improvement Program
VIF: Variance inflation factor.
