# Supplementary material for: Association Between Intoxication and Urgent Neurosurgical Procedures in Severe Traumatic Brain Injury: Results From the American College of Surgeons Trauma Quality Improvement Program
Source: J Intensive Care Med. 2021 May 20;37(3):373–84. doi: 10.1177/08850666211017497 (PMC8772018; doi:10.1177/08850666211017497)
Supplement: Supplemental Material, sj-pdf-1-jic-10.1177_08850666211017497 - Association Between Intoxication and Urgent Neurosurgical Procedures in Severe Traumatic Brain Injury: Results From the American College of Surgeons Trauma Quality Improvement Program [file sj-pdf-1-jic-10.1177_08850666211017497.pdf]

Online-Only Supplementary Material

eTable 1: ICD-9 and -10 codes used to identify neurosurgical procedures

eTable 2: Abbreviated Injury Scale (AIS) 1998 Predot Codes indicating type of intracranial injury

eTable 3: Characteristics of patients who did and did not receive an urgent neurosurgical procedure

eTable 4: Adjusted association between intoxication status and receipt of an urgent neurosurgical procedure

eTables 5: Adjusted association between intoxication status and outcomes stratified by type of neurosurgical procedure

eTables 6: Sensitivity analyses, restricted to patients intoxicated above the legal limit and those tested negative for intoxication

eTables 7: Adjusted association between intoxication status and receipt of an urgent neurosurgical procedure within 4hrs of admission

eTable 1: **ICD-9 and -10 codes used to identify neurosurgical procedures**

	International Statistical Classification of Diseases, Ninth Revision, Clinical Manifestation (ICD-9-CM)	International Statistical Classification of Diseases, Tenth Revision, Clinical Manifestation (ICD-10-CM)
Intracranial pressure monitor	• 01.10<br>• 01.16<br>• 01.17	• 4A000BZ<br>• 4A003BD<br>• 4A003KD<br>• 4A003RD<br>• 4A007BD<br>• 4A007RD
Extra-ventricular Drain	• 02.21	• 0W9130Z<br>• 0W9100Z<br>• Codes starting with:<br>○ 0096<br>○ 0W914<br>○ 0WH13<br>○ 0WH14
Craniotomy	• 01.24	• Codes starting with:<br>○ 0N80<br>○ 0090<br>○ 0091<br>○ 0092<br>○ 0093<br>○ 0094<br>○ 0095<br>○ 0097<br>○ 0099<br>○ 009C<br>○ 00J0<br>○ 0W910Z<br>○ 0WC1<br>○ 0WJ1
Craniectomy	• 01.25<br>• 02.02	• Codes starting with:<br>○ 0N50<br>○ 0NB0<br>○ 0NT1<br>○ 0NT2<br>○ 0NT3<br>○ 0NT4<br>○ 0NT5<br>○ 0NT6<br>○ 0NT7<br>○ 0NT8

eTable 2: **Abbreviated Injury Scale (AIS) 1998 Predot Codes indicating type of intracranial injury**

	AIS 1998 Predot Code
Epidural hematoma	140630, 140632, 140634, 140636
Subdural hematoma	140650, 140652, 140654, 140655, 140656
Traumatic subarachnoid hemorrhage	140684, 140693, 140694, 140695
Intracerebral mass lesion	140602, 140604, 140606, 140608, 140610, 140614, 140616, 140618, 140622, 140624, 140626, 140629, 140638, 140640, 140641, 140646, 140647, 140648, 140649
Compressed/absent basal cisterns	140664, 140666, 140672, 140674
Brainstem/cerebellar lesion	140204, 140206, 140208, 140210, 140212, 140214, 140218, 14218, 140299, 140402, 140403, 140404, 140405, 140406, 140410, 140414, 140418, 140422, 140426, 140430, 140434, 140438, 140442, 140446, 140450, 140458, 140462, 140466, 140470, 140472, 140473, 140474, 140489, 140499

eTable 3: **Characteristics of patients who did and did not receive an urgent neurosurgical procedure**

	Overall (n =33,646)	Received an urgent neurosurgical procedure (n= 8,255)	No urgent neurosurgical procedure (n= 25,391)	Standardized difference
Patient Characteristics				
Age, median (IQR)	51 (31 – 68)	47 (29 – 63)	52 (31 – 70)	0.20
Age group, n (%)				
16-40	12,478 (37.1)	3,414 (41.3)	9,064 (35.7)	
41-64	11,326 (33.7)	2,969 (36.0)	8,357 (32.9)	0.18
65-89	9,824 (29.3)	1,872 (22.7)	7,970 (31.4)	
Female, n (%)	9,438 (28.1)	2,140 (25.9)	7,298 (28.7)	0.06
Race, n (%)				
Non-Hispanic white	22,683 (67.4)	5,389 (65.3)	17,294 (68.1)	
Hispanic or Latino	3,806 (11.3)	1,090 (13.2)	2,716 (10.7)	0.10
Black	3,524 (10.5)	837 (10.1)	2,687 (10.6)	
Other minority group	2,417 (7.2)	606 (7.3)	1,811 (7.1)	
No. comorbid illnesses, n (%)				
0	11,440 (34.0)	2,965 (35.9)	8,475 (33.4)	
1	11,251 (33.4)	2,794 (33.9)	8,457 (33.3)	0.10
2	6,177 (18.4)	1,408 (17.1)	4,769 (18.8)	
≥ 3	4,778 (14.2)	2,965 (35.9)	8,475 (33.4)	
Type of insurance, n (%)				
Commercial	10,642 (31.6)	2,910 (35.3)	7,732 (30.5)	
Non-commercial	15,580 (46.3)	3,614 (43.8)	11,966 (47.1)	0.11
Self-pay	4,569 (13.6)	1,052 (12.7)	3,517 (13.9)	
Other	1,233 (3.7)	266 (3.2)	967 (3.8)	
Intoxicated, n (%)	11,313 (33.6)	2,878 (34.9)	8,435 (33.2)	0.03
Intoxication category, n (%)				
Confirmed not intoxicated	14,005 (41.6)	3,569 (43.2)	10,436 (41.1)	
Not tested assumed not intoxicated	8,328 (24.8)	1,808 (21.9)	6,520 (25.6)	
Not tested assumed intoxicated	843 (2.5)	222 (2.7)	621 (2.5)	0.11
Intoxicated below the legal limit	2,083 (6.2)	588 (7.1)	1,495 (5.9)	
Intoxicated above the legal limit	8,387 (24.9)	2,068 (25.1)	6,319 (24.9)	
Injury Characteristics				
Mechanism of injury, n (%)				
Fall	17,441 (51.8)	3,998 (48.4)	13,443 (52.9)	
MVC	5,614 (16.7)	1,337 (16.2)	4,277 (16.8)	
Motorcycle	2,037 (6.1)	535 (6.5)	1,502 (5.9)	0.12
Pedestrian	2,004 (6.0)	580 (7.0)	1,424 (5.6)	
Cyclist	997 (3.0)	289 (3.5)	708 (2.8)	
Other	5,372 (16.0)	1,473 (17.8)	3,899 (15.4)	
AIS head, n (%)				
3	6,613 (19.7)	581 (7.0)	6,032 (23.8)	
4	11,491 (34.2)	2,416 (29.3)	9,075 (35.7)	0.58
5	15,542 (46.2)	5,258 (63.7)	10,284 (40.5)	
Type of head injury, n (%)				
EDH	3,012 (9.0)	1,428 (17.3)	1,584 (6.2)	0.35
SDH	20,290 (60.3)	5,905 (71.5)	14,385 (56.7)	0.31
Traumatic SAH	16,184 (48.1)	4,087 (49.5)	12,097 (47.6)	0.04
Intracerebral mass lesion	10,838 (32.2)	2,871 (34.9)	7,967 (31.4)	0.07

	Overall (n =33,646)	Received an urgent neurosurgical procedure (n= 8,255)	No urgent neurosurgical procedure (n= 25,391)	Standardized difference
Compressed basal cisterns	2,026 (6.0)	767 (9.3)	1,259 (5.0)	0.17
Brainstem/cerebellar lesion	3,402 (10.1)	960 (11.6)	2,442 (9.6)	0.07
Other brain injury without any of the above	3,486 (10.4)	344 (4.2)	3,142 (12.4)	0.30
ISS, median (IQR)	24 (16 – 26)	25 (20 – 27)	21 (16 – 25)	0.53
ISS, n (%)				
9-15	5,420 (16.1)	440 (5.3)	4,980 (19.6)	0.58
16-24	12,680 (37.7)	2,556 (31.0)	10,124 (39.9)	
25-75	15,546 (46.2)	5,259 (63.7)	10,287 (40.5)	
Minor injury in another body region, n (%)	19,062 (56.7)	4,536 (55.0)	14,526 (57.2)	0.05
Hypotension (SBP <90), n (%)	1,302 (3.9)	251 (3.0)	1,051 (4.1)	0.06
Motor GCS, n (%)				
1	22,090 (65.7)	5,361 (64.9)	16,729 (65.9)	0.10
2-3	3,228 (9.6)	963 (11.7)	2,265 (8.9)	
4-5	8,053 (23.9)	1,861 (22.5)	6,192 (24.4)	
Hospital Characteristics				
Teaching status, n (%)				
University	19,124 (56.8)	4,633 (56.1)	14,491 (57.1)	0.02
Community	11,590 (34.5)	2,915 (35.3)	8,675 (34.2)	
Non-teaching	2,932 (8.7)	707 (8.6)	2,225 (8.8)	
Bed size, n (%)				
≤ 200	1,004 (3.0)	282 (3.4)	722 (2.8)	0.02
201-400	7,158 (21.3)	1,847 (22.4)	5,311 (20.9)	
401-600	10,088 (30.0)	2,383 (28.9)	7,705 (30.4)	
> 600	15,396 (45.8)	3,743 (45.3)	11,653 (45.9)	
Trauma center level, n (%)				
I	23,244 (69.1)	5,717 (69.3)	17,527 (69.0)	<0.01
II	9,811 (29.2)	2,359 (28.6)	7,452 (29.4)	
Other	591 (1.8)	179 (2.2)	412 (1.6)	
No. neurosurgeons, n (%)				
≤ 2	2,433 (7.2)	660 (8.0)	1,773 (7.0)	0.04
3-5	15,159 (45.1)	3,726 (45.1)	11,433 (45.0)	
≥ 6	16,054 (47.7)	3,869 (46.9)	12,185 (48.0)	

IQR = interquartile range; AIS = abbreviated injury scale; EDH = epidural hematoma; SDH = subdural hematoma; SAH = subarachnoid hemorrhage; ISS = injury severity score; SBP = systolic blood pressure; GCS = Glasgow Coma Scale; MVC = motor vehicle collision

eTable 4: **Adjusted association between intoxication status and receipt of an urgent neurosurgical procedure**

Predictor Variables	Adjusted OR (95% CI)	p-value
Intoxicated	0.99 (0.94 – 1.06)	0.96
Age, per year increase		
Among those below age 65	0.99 (0.99 – 1.00)	<0.001
Among those age 65 and older	0.95 (0.94 – 0.96)	
Female	0.99 (0.92 – 1.05)	0.68
Race		
Non-Hispanic white	Ref	<0.001
Hispanic or Latino	1.24 (1.15 – 1.37)	
Black	1.00 (0.91 – 1.10)	
Other minority group	0.99 (0.89 – 1.11)	
Number of Comorbid Illnesses		
0	REF	0.51
1	1.03 (0.96 – 1.11)	
2	0.99 (0.91 – 1.08)	
≥ 3	0.97 (0.88 – 1.07)	
AIS Head		
3	REF	<0.001
4	2.07 (1.85 – 2.32)	
5	4.29 (3.84 – 4.80)	
Epidural hematoma	2.33 (2.13 – 2.54)	<0.001
Subdural hematoma	1.39 (1.29 – 1.49)	<0.001
Traumatic SAH	1.00 (0.94 – 1.06)	0.99
Intracerebral mass lesion	1.06 (1.00 – 1.13)	0.05
Compressed/absent basal cisterns	1.20 (1.08 – 1.34)	<0.001
Brainstem/cerebellar lesion	1.06 (0.96 – 1.16)	0.25
Other brain injury	0.59 (0.51 – 0.68)	<0.001
Minor Injury in Another Body Region	0.88 (0.83 – 0.93)	<0.001
Hypotension	0.75 (0.64 – 0.88)	<0.001
Motor GCS		
5	REF	<0.001
4	1.01 (0.90 – 1.14)	
3	1.30 (1.12 – 1.50)	
2	1.20 (1.03 – 1.40)	
1	0.97 (0.88 – 1.08)	
Mechanism of Injury		
MVC	REF	<0.001
Fall	0.89 (0.81 – 0.98)	
Motorcycle	1.07 (0.94 – 1.22)	
Pedestrian	1.16 (1.02 – 1.33)	
Cyclist	1.13 (0.96 – 1.35)	
Other	1.10 (1.00 – 1.22)	
Type of Insurance		
Commercial	REF	<0.001
Non-Commercial	0.95 (0.89 – 1.02)	
Self-pay	0.76 (0.69 – 0.83)	
Other	0.72 (0.62 – 0.84)	
Type of Hospital		
University	REF	0.13
Community	1.07 (1.00 – 1.16)	

Non-teaching	1.09 (0.97 – 1.23)	
Bed Size		
> 600	REF	
401-600	0.91 (0.85 – 0.97)	<0.001
201-400	1.04 (0.95 – 1.13)	
≤ 200	1.28 (1.09 – 1.51)	
Level I Trauma Centre	1.09 (1.00 – 1.18)	0.04
Number of Neurosurgeons		
≥ 6	REF	
3-5	1.04 (0.98 – 1.11)	0.02
≤ 2	1.17 (1.05 – 1.31)	

eTable 5: Adjusted association between intoxication status and outcome stratified by type of neurosurgical procedure

Outcome	Subgroup	Proportion intoxicated who experienced outcome, n (%)	Association between intoxication and outcome, adjusted odds ratio (95% CI)	p-value
**Receipt of an urgent procedure**				
ICP monitor		1,717 (15.2)	1.01 (0.93 – 1.08)	0.89
	No significant subgroup			
Craniotomy		1,219 (10.8)	0.97 (0.89 – 1.06)	0.52
	AIS head			0.009
	3	64 (2.4)	0.62 (0.44 – 0.88)	
	4	296 (7.1)	0.90 (0.76 – 1.05)	
	5	859 (19.4)	1.05 (0.94 – 1.16)	
Craniectomy		703 (6.2)	1.00 (0.90 – 1.11)	0.98
	No significant subgroup			
**Receipt of a timely urgent procedure**				
ICP monitor		1,263 (73.6)	0.88 (0.76 – 1.03)	0.11
	No significant subgroup			
Craniotomy		903 (74.1)	0.98 (0.82 – 1.18)	0.83
	No significant subgroup			
Craniectomy		519 (73.8)	0.89 (0.69 – 1.15)	0.37
	No significant subgroup			

ICP = intracranial pressure

eTable 6: **Sensitivity analyses, restricted to patients intoxicated above the legal limit and those tested negative for intoxication**

Outcome	Subgroup	Proportion intoxicated who experienced outcome, n (%)	Association between intoxication and outcome, adjusted odds ratio (95% CI)	p-value
**Receipt of an urgent procedure**				
Any neurosurgical procedure		2,068 (24.7)	0.89 (0.83 – 0.96)	0.002
	AIS head			0.01
	3	165 (7,6)	0.70 (0.56 – 0.87)	
	4	649 (20.9)	0.84 (0.74 – 0.95)	
	5	1,254 (40.3)	0.97 (0.88 – 1.08)	
ICP monitor	No significant subgroup	1,260 (15.0)	0.89 (0.81 – 0.97)	0.007
Craniotomy	No significant subgroup	881 (10.5)	0.94 (0.84 – 1.04)	0.21
Craniectomy	No significant subgroup	499 (6.0)	0.87 (0.77 – 1.00)	0.04
**Receipt of a timely urgent procedure**				
Any neurosurgical procedure	No significant subgroup	1,520 (73.5)	0.93 (0.81 – 1.07)	0.31
ICP monitor	No significant subgroup	923 (73.3)	0.86 (0.72 – 1.03)	0.09
Craniotomy	No significant subgroup	639 (72.5)	0.88 (0.71 – 1.10)	0.26
Craniectomy	No significant subgroup	370 (74.2)	0.89 (0.66 – 1.20)	0.43

ICP = intracranial pressure

eTables 7: **Adjusted association between intoxication status and receipt of an urgent neurosurgical procedure within 4hrs of admission**

**Predictor Variables**	**Adjusted OR (95% CI)**	**p-value**
Intoxicated	0.98 (0.87 – 1.10)	0.69
Age, per year increase	1.00 (1.00 – 1.00)	0.57
Female	1.02 (0.90 – 1.17)	0.73
Race		
Non-Hispanic white	Ref	<0.001
Hispanic or Latino	0.88 (0.75 – 1.03)	
Black	0.66 (0.55 – 0.78)	
Other minority group	0.95 (0.76 – 1.17)	
Number of Comorbid Illnesses		
0	REF	0.009
1	0.99 (0.86 – 1.13)	
2	0.77 (0.66 – 0.91)	
≥ 3	0.93 (0.77 – 1.13)	
AIS Head		
3	REF	<0.001
4	1.19 (0.96 – 1.47)	
5	1.96 (1.58 – 2.43)	
Epidural hematoma	1.23 (1.05 – 1.44)	0.01
Subdural hematoma	1.23 (1.07 – 1.41)	0.004
Traumatic SAH	0.72 (0.64 – 0.81)	<0.001
Intracerebral mass lesion	0.75 (0.66 – 0.84)	<0.001
Compressed/absent basal cisterns	1.17 (0.96 – 1.44)	0.12
Brainstem/cerebellar lesion	0.91 (0.76 – 1.09)	0.30
Other brain injury	0.64 (0.48 – 0.85)	0.002
Minor Injury in Another Body Region	0.76 (0.67 – 0.86)	<0.001
Hypotension	1.36 (0.97 – 1.91)	0.07
Motor GCS		
5	REF	<0.001
4	1.07 (0.85 – 1.33)	
3	1.01 (0.77 – 1.33)	
2	1.58 (1.16 – 2.15)	
1	1.31 (1.08 – 1.58)	
Mechanism of Injury		
MVC	REF	<0.001
Fall	1.34 (1.12 – 1.59)	
Motorcycle	0.94 (0.75 – 1.19)	
Pedestrian	1.20 (0.95 – 1.52)	
Cyclist	1.17 (0.85 – 1.60)	
Other	1.44 (1.20 – 1.73)	
Type of Insurance		
Commercial	REF	0.66
Non-Commercial	1.06 (0.93 – 1.21)	
Self-pay	1.11 (0.93 – 1.32)	
Other	1.03 (0.76 – 1.40)	
Type of Hospital		
University	REF	0.02
Community	1.21 (1.05 – 1.40)	
Non-teaching	1.28 (1.00 – 1.63)	
Bed Size		

> 600	REF	0.04
401-600	1.13 (0.99 – 1.29)	
201-400	1.28 (1.08 – 1.52)	
≤ 200	1.17 (0.85 – 1.61)	
Level I Trauma Centre	1.04 (0.88 – 1.22)	0.68
Number of Neurosurgeons		
≥ 6	REF	0.98
3-5	1.01 (0.89 – 1.14)	
≤ 2	0.99 (0.79 – 1.23)	

ISS and total GCS score were excluded from the final model as they were colinear with AIS and motor GCS.
